# Effect of arthroscopic acromioplasty on reducing critical shoulder angle: a protocol for a prospective randomized clinical trial

**DOI:** 10.1186/s12891-020-03818-w

**Published:** 2020-12-07

**Authors:** Yi Long, Jingyi Hou, Yiyong Tang, Fangqi Li, Menglei Yu, Congda Zhang, Rui Yang

**Affiliations:** Department of Orthopedics, Sun Yat-sen Memorial Hospital, Sun Yat-sen University, 107 Yan Jiang Road West, Guangzhou, 510120 Guangdong China

**Keywords:** Critical shoulder angle, Acromioplasty, Rotator cuff tears, 3D reconstruction

## Abstract

**Background:**

The critical shoulder angle (CSA), which helps to predict patients who are at risk of rotator cuff tears (RCTs) with large degree and who are susceptible to osteoarthritis with low angle, has been identified as one of the most vital acromial parameters; anterolateral and lateral acromioplasties have been proven to be valid ways to reduce CSA. However, no study has compared the effect of different acromioplasties on the reduction of the large CSA (≥33°) clinically. Additionally, either anterolateral or lateral acromioplasty could not precisely correct large CSAs to a favorable range (30–33°) in each patient. Thus, we will propose a novel precise acromioplasty technique for the purpose of reducing CSA accurately and effectively, and compare the effectiveness of different acromioplasties on the reduction of the CSA.

**Methods:**

A total of 60 RCT patients who have indications for arthroscopic rotator cuff repair and with pre-operative CSA ≥33° will be recruited in outpatient center of Sun Yat-sen Memorial Hospital. Eligible participants will be randomly allocated to Group A (anterolateral acromioplasty), Group B (lateral acromioplasty) or Group C (precise acromioplasty) via a random, computer-generated number system. Three surgical plans will be made for each participant respectively by one professional surgeon according to the results of randomization allocation. The post-operative CSA will be measured 2 days post-operation. Follow-up will be maintained at 3, 6, and 12 months after surgery including the visual analog scale score, the University of California at Los Angeles score, the Constant Shoulder Score and the American Shoulder and Elbow Surgeon Shoulder Assessment Form. Finally, all outcomes will be assessed by two researchers who are blinded to the recruitment and allocation.

**Discussion:**

This is the first clinical trial to evaluate the impact of different acromioplasties on the reduction of the CSA. Additionally, this study will provide a new precise acromioplasty technique, which is a novel precision and individualized treatment to prevent degenerative RCTs by reducing the CSA.

**Trial registration:**

ChiCTR2000032343. Registered on April 26th, 2020.

## Background

Rotator cuff tears (RCTs) is a common shoulder disease in the general adult population [1, 2]. While the pathogenesis of degenerative RCTs are multi-faceted, the precise mechanisms are still not fully understood [3–5]. The acromial morphology has been identified as one of the important etiologic factors of degenerative RCTs [6–8]. Numerous studies have revealed that a type III acromion, higher acromion index, lower lateral acromion angles, and larger critical shoulder angles (CSAs) are significantly associated with degenerative RCTs [9–11].

The CSA, defined as the angle between the plane of the glenoid fossa and a line connecting the inferolateral point of the acromion with the inferior glenoid margin on standardized anterior radiographs [12], has been identified as one of the most accurate and vital anatomic predictors of the development of degenerative RCTs [13]. Several studies have documented that a CSA of more than 35° predicts RCTs, whereas a CSA of 30° or less is associated with glenohumeral osteoarthrtitis (OA) or superior labrum from anterior to posterior lesion (SLAP) [14–19]. Gerber et al. concluded that correcting a large CSA to 33° or less during arthroscopic rotator cuff repair (RCR) could help patients to achieve superior strength of abduction after the RCTs heal [20]. Similarly, we previously found that a reduction of a large CSA (≥33°) to a desired range (30–33°) may prevent RCTs and OA [21].

Arthroscopic anterolateral or lateral acromioplasty has been proven to change the pathologically increased CSA in cadaveric study [22, 23]. Some authors also reported that the CSA can be reduced significantly by anterolateral or lateral acromioplasty in vivo [20, 24, 25]. Furthermore, Kaiser et al. demonstrated that lateral acromioplasty decreased the CSA more significantly than anterolateral acromioplasty in a three-dimensional analysis [26]. Nevertheless, to our knowledge, no study has yet compared the effect of different arthroscopic acromioplasties on the reduction of the large CSA (≥33°) clinically. Besides, although these techniques can greatly reduce CSA, none precisely reduce large CSAs to the favorable range.

The primary goal of the current study is to propose the precise acromioplasty, which is a novel technique for the purpose of reducing the CSA sufficiently and accurately. The second goal is to conduct a prospective randomized clinical trial that will evaluate the effectiveness of different acromioplasties on the reduction of CSA.

## Methods

### Study design

**Fig. 1:**
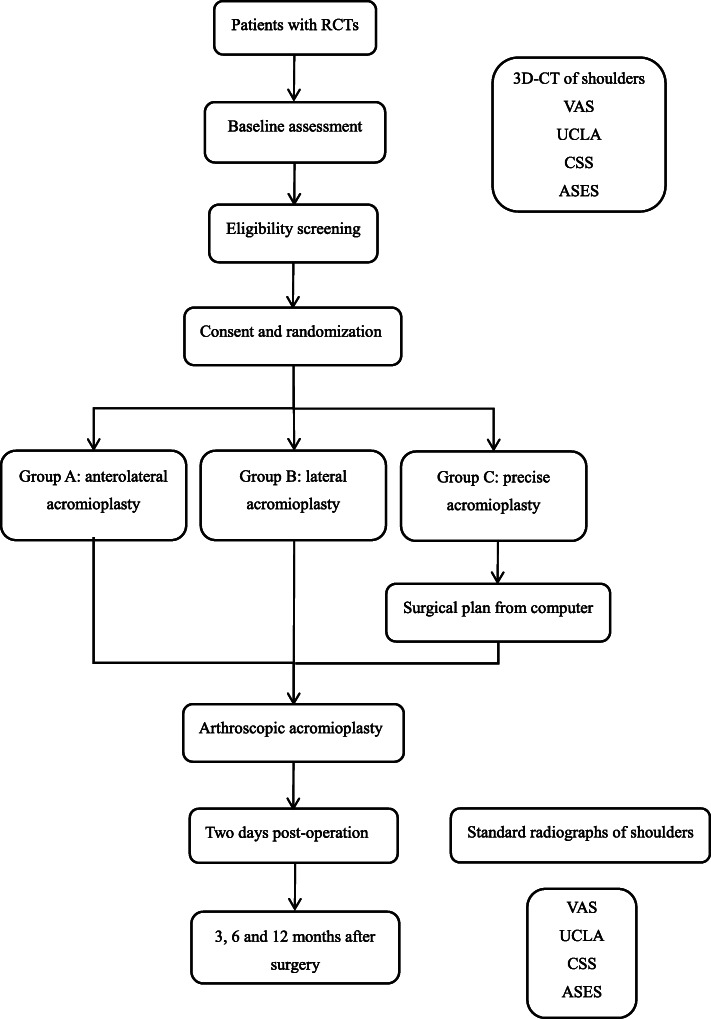
Study flow diagram

This study is a prospective, single-center, parallel-design randomized controlled trial. This protocol has been developed according to the SPIRIT statement [27]. The trial has been prospectively registered in the Chinese Clinical Trial Registry (ChiCTR2000032343). The outline of the study was given in Fig. 1.

### Recruitment and informed consent

This study will be conducted in Sun Yat-sen Memorial Hospital. Patients with rotator cuff tears (RCTs) who need arthroscopic rotator cuff repair (RCR) will be recruited from the outpatient center of Sun Yat-sen Memorial Hospital. After accomplishing baseline assessment and confirming the eligibility of the patients, the researcher will introduce the study purposes, procedures, possible risks etc. for approximately 45 min, and answer all the questions raised by patients and their families. Informed consent forms will be signed by each patient. After enrollment, participants will be coded with a unique number.

### Eligibility and exclusion criteria

The inclusion criteria are as follows: diagnosis of unilateral degenerative RCTs; pre-operative critical shoulder angle (CSA) ≥33°; required to receive arthroscopic RCR; willing to take part in this study; pre-operative three-dimensional computed tomography (3D-CT) and post-operative standard radiographs of shoulder were performed in our institution. The exclusion criteria are: history of shoulder trauma, and concomitant fracture or dislocation of shoulder; previous surgery of the involved shoulder; diagnosis of massive rotator cuff tears; concomitant frozen shoulder, glenohumeral osteoarthrtitis (OA) or other inflammatory arthritis; unable to give informed consent.

### Randomization

After informed consent, eligible participants will receive a unique number. Then, patients will be randomly allocated to either the anterolateral acromioplasty group (Group A), the lateral acromioplasty group (Group B), or precise acromioplasty group (Group C) via a random, computer-generated number system. The treating surgeon will determine surgical plans according to the results of the randomization allocation. Allocation will be based on a 1:1:1 ratio between the three different groups.

### Blinding

In this study, participants will be clearly informed of surgical plans before operation. Besides, the surgeon and assistant will perform acromioplasty along with arthroscopic RCR. Consequently, the patients, treating surgeon and assistant are not blinded to the group allocation. However, the surgeon and assistant will not participate in the pre-operative and post-operative assessment, and other researchers will blinded to the surgical decision making.

### Interventions

All patients will receive a routine examination during admission, including routine blood and urine tests, as well as evaluation of general condition. Imaging examination, such as a 3D-CT of shoulder and MRI (if necessary), will be completed before enrollment. All participants will complete standard anteroposterior radiography of shoulders 2 days post-operation and follow-up will be continued at 3, 6 and 12 months after surgery. This trial will involve three types of acromioplasty: anterolateral, lateral or precise acromioplasty. One of these acromioplasties will be randomly performed along with arthroscopic RCR in each group.

### Surgical procedures

One professional surgeon will make the surgical plan and conduct the arthroscopy in Sun Yat-sen Memorial Hospital. All participants will undergo general anaesthesia and be fixed in lateral decubitus. The surgery will begin with the establishment of a standard posterior portal and diagnostic arthroscopy. Then the arthroscopic RCR will be performed depending on the tear location and size. Later, three different acromioplasties will be conducted in groups A, B and C respectively according to a random allocation as previously described. The main processes of the acromioplasties are described below.

In group A, after the undersurface of the acromioclavicular joint and the anterolateral margin of the acromion are freed from all soft tissue via a 5 mm shaver burr (Arthrex Inc., Naples, USA), the surgeon will gradually resect the anterolateral acromion from the anterolateral corner, perpendicular to the line from the anterior third lateral edge of the acromion to anterior acromioclavicular joint line margin. The anterolateral acromioplasty in group A will involve a width of approximately 10 mm (two shaver burr widths).

In group B, after the undersurface and lateral margin of the acromion are freed from all soft tissue, the lateral acromioplasty will be started inferiorly at the middle lateral edge of the acromion, perpendicular to the line from the anterolateral to the posterolateral corner of the acromion. The lateral acromioplasty in group B will involve a width of approximately 10 mm (two shaver burr widths).

**Fig. 2:**
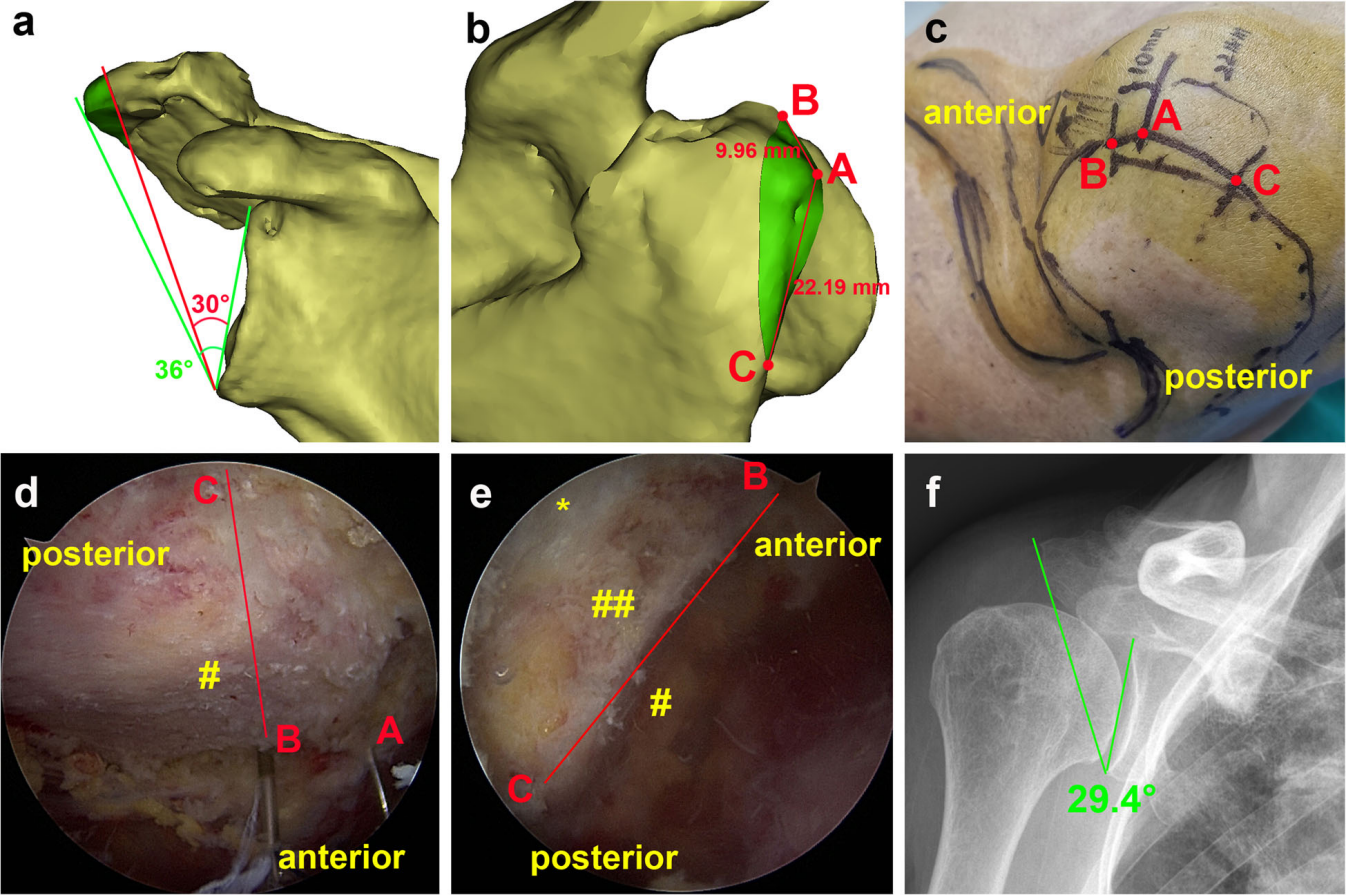
The procedure of the precise acromioplasty. **a** Simulating virtual acromioplasty on computer. The pre-operative CSA (critical shoulder angle) is 36°(green line), the post-operative target CSA is set to be 30°(red line), and the green regions is planned to be resected. **b** Measurement of the resected bone border (green regions) by software. **A**, anterolateral corner; **B**, anteromedial resection margin; **C**, posterolateral resection margin; distance from **A** to **B** is about 10 mm, distance from **A** to **C** is about 22 mm. **c** Marking acromion and determining the resection margin on the patient’s skin. **d** View from the posterior portal: The spinal needles mark the points **A, B** and **C**, the line BC is the resection line. # undersurface of the acromion. **e** View from the lateral portal: After the precise resection of the acromion. * delta muscle fibers; ## resection surface of the acromion. **f** Measurement of post-operative CSA (29.4°) on standard anteroposterior radiography of the shoulder

In group C, the pre-operative CSA (≥33°) will be measured on the 3D model. We will set 30° for the CSA as the post-operative target degree. Second, the virtual acromioplasty will be simulated and the resected bone border will be measured upon the Mimics (Materialise, Leuven, Belgium), including the anterolateral corner of acromion (A), anteromedial resection margin (B), posterolateral resection margin (C), and the distance of AB and AC. Third, the surgeon will identify and mark points A, B and C on the patient’s skin by measurement the distance of AB and AC according to the pre-operative planning above. Then, after the undersurface of the acromion are freed from all soft tissue, the surgeon will insert points A, B and C with spinal needles perpendicularly from the skin to the subacromial space as line marks, a resection line will be made from B to C during arthroscopy. Lastly, the region of the acromion outside the resection line will be gradually resected by a 5 mm shaver burr (Arthrex Inc., Naples, FL, USA) during arthroscopy. The main procedure is shown schematically in Fig. 2.

### Outcome measures

General information and baseline assessment will be collected during admission. The post-operative CSA will be measured 2 days post-operation. Follow-up will be maintained at 3, 6, and 12 months after surgery, including the visual analog scale (VAS) score, the University of California at Los Angeles (UCLA) score, the Constant Shoulder Score (CSS) and the American Shoulder and Elbow Surgeon (ASES) Shoulder Assessment Form. All outcomes will be assessed by two researchers who are blinded to the recruitment and allocation.

### Primary outcome

#### Pre-operative CSA on 3D scapula

**Fig. 3:**
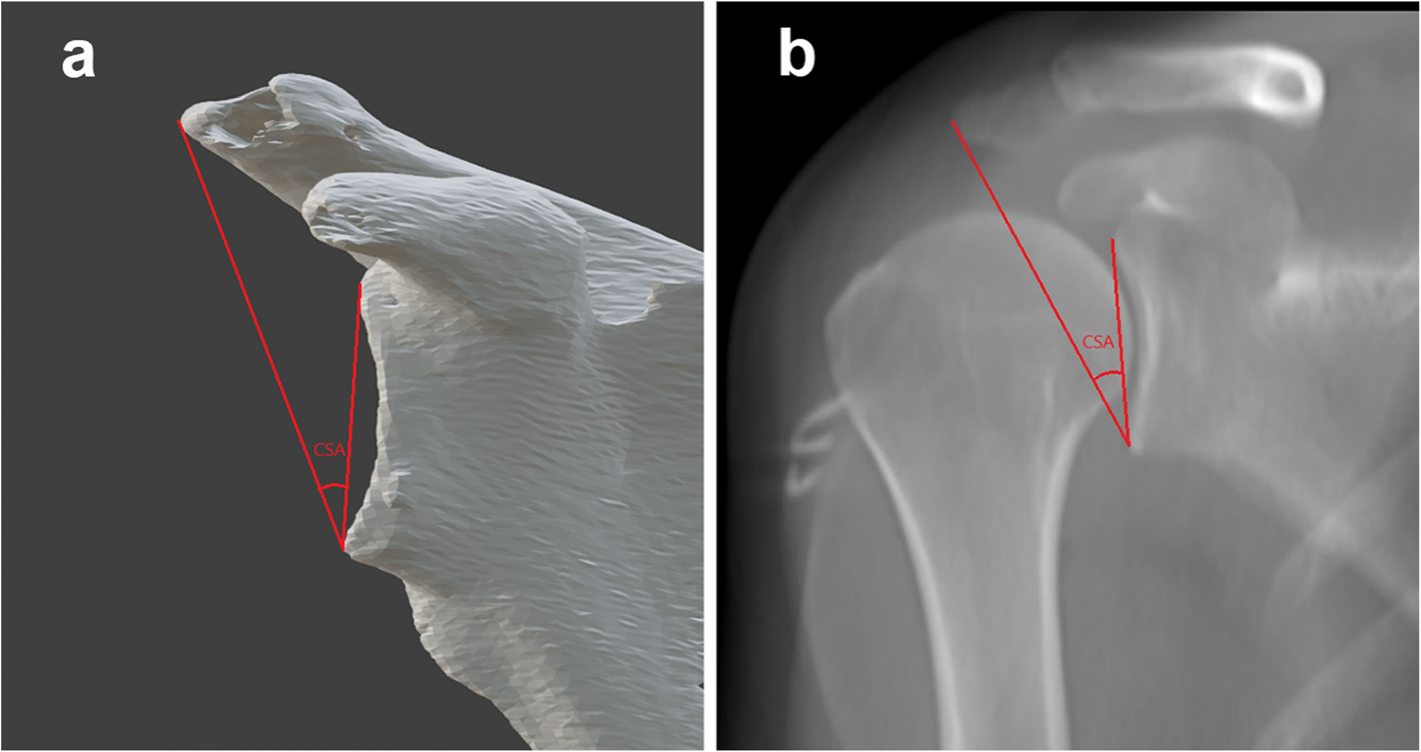
The measurement of the critical shoulder angle (CSA) on 3D scapula (**a**) and X-ray (**b**)

The pre-operative CSA of patients will be measured on 3D-CT model before enrollment. First, Digital Imaging and Communications in Medicine (DICOM) data from CT scans of the shoulders will be used to reconstruct 3D scapulae by Mimics (Materialise, Leuven, Belgium). Then, the 3D model will be imported into Blender (Amsterdam, Netherlands) and aligned to the local coordinate system according to the same criteria as described by Suter et al. [28]. Lastly, the CSA, which is measured as the angle between a line connecting the inferior border of the glenoid with the most inferolateral point of the acromion and another connecting inferior and superior glenoid margin [12], will be measured on anterior view using Blender (Fig. 3a). Patients with a native CSA ≥33° will be recruited as candidates.

#### Post-operative CSA on X-ray

The post-operative CSA of participants will be measured on standard anteroposterior radiography of the shoulder (Fig. 3b). The efficacy is reflected in the reduction of CSA after acromioplasty. The accuracy is defined as the percentage of patients whose post-operative CSA decreased from a large angle (≥33°) to a normal range (30–33°) via acromioplasty.

### Secondary outcome

#### Shoulder function scores

Subjective pain will be ranked by the patients on the visual analog scale (VAS) score for pain, with 0 indicating no pain at all and 10 indicating the most severe pain the patient could imagine. The University of California at Los Angeles (UCLA) score, the Constant Shoulder Score (CSS) and the American Shoulder and Elbow Surgeon (ASES) Shoulder Assessment Form will be used for the functional assessment.

### Adverse event management

In this study, the different interventions for participants who are willing to receive arthroscopic RCR are three diverse acromioplasties, which are relatively safe procedures during minimally invasive surgery. Adverse events are defined as any unexpected medical incidents which happen in participants and do not necessarily have a causality with the acromioplasty, including acromial fractures, detachment of deltoid origin, and either pain or swelling due to bone removal of acromion. Participants will report any adverse events to doctors or study researchers as soon as they can. All adverse events will be documented and further medical treatment will be arranged if necessary. In addition, the participants will be closely followed up until conditions are resolved or their condition is stabilized.

### Data management

Participants will be coded with a study number after informed consent, and all data referring to patients will be documented by this number, not by name. A case report form (CRF) will be used for participants to collect information including baseline information, radiographic outcomes, and follow-up. Data will be collected at baseline admission and 2 days post-operation, as well as 3, 6 and 12 months post-surgery. Data on adverse events and further medical treatments will also be collected. The recording of data is assigned to a data manager who is accountable for it, and other researchers will be restricted from accessing study data.

### Sample size calculation

The primary outcome of this trial is the CSA and a power analysis was performed using PASS 15 (NCSS LLC, USA) to calculate the sample size. According to the results of Kaiser’s study [26] and our preliminary study, the reduction of CSA was 2.6° ± 1.8° (mean ± standard deviation, SD) in group A, 4.4° ± 1.5° in group B, and 4.8° ± 1.6° in group C respectively. Based on a power of 90%, and an alpha error of 0.05, a sample size of 48 patients was determined. After taking into consideration a dropout rate of 20%, a sample of 60 participants (20 participants per group) will be recruited.

### Statistical analysis

The analysis of covariance will be conducted to compare the alteration of the CSA and shoulder function scores between randomized groups. The accuracy rates of different acromioplasties will be calculated, and χ^2^ test will be performed to evaluate the statistical differences between groups. The Spearman correlation coefficients will be used to assess the relationship between CSA and shoulder function scores. The intraclass correlation coefficient will be calculated to assess the consistency between researchers and between measurements of a single researcher. Statistical significance will be set at *P* < 0.05. All analyses will be carried out using SPSS 19 (IBM Corp, USA) by two researchers blinded to the recruitment and collection.

## Discussion

The critical shoulder angle (CSA), which combines the measurements of the lateral extension of the acromion and the inclination of the glenoid joint plane, was originally introduced by Moor et al. [12]. They documented that degenerative rotator cuff tears (RCTs) had significantly larger CSAs (≥35°) than asymptomatic shoulders without this pathology, and that a CSA smaller than 30° is associated with glenohumeral osteoarthrtitis (OA). Gerber et al. confirmed these conclusions through a biomechanical analysis which demonstrated that a high CSA could induce supraspinatus (SSP) overload and that a low CSA increased the pressure of the humeral head on the glenoid [29–31]. To date, altering the CSA by arthroscopic acromioplasty is a common strategy. In 2017, Gerber et al. first demonstrated that the mean CSA was significantly corrected from 37.5° pre-operatively to 33.9° post-operatively through lateral acromioplasty in vivo [20]. Billaud et al. first reported in a clinical study that the average CSA decreased from 35.9° to 33° after anterolateral acromioplasty (anterior third of the acromion to be resected) [24]. Although arthroscopic acromioplasty is an effective procedure to decrease CSAs, questions remain regarding the necessity of acromioplasty performed alongside rotator cuff repair (RCR) has never ceased [32]. Several studies have found no difference in patient-reported outcome scores for patients who had arthroscopic RCR with or without acromioplasty [33–35]; besides, the reduction of the CSA does not improve functional results postoperatively [36, 37]. Conversely, some authors have demonstrated that large CSAs increase the risk of re-tearing after RCR [38–41]. Gerber et al. showed that abnormally large CSAs are associated either with higher re-tear rates or with inferior strength of abduction after reconstruction [20]. As most of these authors suggested, we recommend that if the CSA is greater than 33°, acromioplasty should be performed during arthroscopic RCR to reduce large CSAs to normal range (30–33°) [21].

Although lateral acromioplasty has been documented to be the most effective way to decrease CSAs in vitro [22, 26], the post-operative angle is uncontrollable due to the insufficient or over-resection of the acromion. In other words, this is not always correct the large CSAs into the desired range of 30–33°. Additionally, lateral acromioplasty up to 10 mm has been considered a safe technique [20, 22, 42], yet some authors reported that over-resection of the acromion has potential complications, such as detachment of the deltoid origin and acromial fractures [43–45]. In 2017, Karns et al. introduced the concept of the critical acromion point (CAP), which is the most inferolateral part of the acromion and the key point used to determine the degree of the CSA on true anteroposterior radiograph. They also proposed a virtual acromioplasty on three-dimensional (3D) scapulae models with resection of the acromion parallel to the glenoid face of 2.5/5 mm medial to the CAP [46]. This technique seemed to have the equivalent effect in altering the CSA while preserving more bone volume when compared with lateral acromioplasty. However, only 80% of specimens with a native CSA of 38° or greater could be decreased to the acceptable range by this virtual acromioplasty; in addition, this technique has not been clinically realized so far.

To addresses the aforementioned deficiencies, we developed a precise acromioplasty technique, which is an individualized therapeutic strategy oriented by pre-operative virtual acromioplasty on 3D-CT model and software analysis, for the purpose of reducing the CSA with greater efficacy and accuracy. Theoretically, we can clinically decrease large CSAs to any desired specific angle by this technique without cutting acromion not contributing to the CSA. Additionally, we will conduct a prospective randomized clinical trial to examine patients with RCTs and a CSA of 33° or greater who undergo acromioplasty during arthroscopic RCR to evaluate the impact of different acromioplasties. This study will provide a new precision and individualized treatment to reduce CSA.

## Data Availability

The results of this trial will be made available upon request by the corresponding author.

## Abbreviations

ASES: American Shoulder and Elbow Surgeon Shoulder Assessment Form
BMI: Body mass index
CAP: Critical acromion point
CRF: Case report form
CSA: Critical shoulder angle
CSS: Constant Shoulder Score
DICOM: Digital Imaging and Communications in Medicine
MRI: Magnetic resonance imaging
OA: Osteoarthritis
RCR: Rotator cuff repair
RCTs: Rotator cuff tears
SLAP: Superior labrum from anterior to posterior lesion
SSP: Supraspinatus
3D-CT: Three-dimensional computed tomography
UCLA: University of California at Los Angeles score
VAS: Visual analog scale score
